# Effects of stimulating interleukin -2/anti- interleukin -2 antibody complexes on renal cell carcinoma

**DOI:** 10.1186/s12894-016-0121-2

**Published:** 2016-01-16

**Authors:** Kyu-Hyun Han, Ki Won Kim, Ji-Jing Yan, Jae-Ghi Lee, Eun Mi Lee, Miyeon Han, Eun Jin Cho, Seong Sik Kang, Hye Jin Lim, Tai Yeon Koo, Curie Ahn, Jaeseok Yang

**Affiliations:** Transplantation Research Institute, Seoul National University College of Medicine, Seoul, Republic of Korea; Nephrology clinic, Center for Clinical Specialty, National Cancer Center, Seoul, Republic of Korea; Department of Internal Medicine, Seoul National University College of Medicine, Seoul, Republic of Korea; Transplantation Center, Seoul National University Hospital, 101 Daehak-ro, Jongno-gu, Seoul, 110-744 Republic of Korea

**Keywords:** CD8^+^ T cell, Immune complex, Interleukin-2, NK cell, Renal cell carcinoma, Tumor

## Abstract

**Background:**

Current therapies for advanced renal cell carcinoma (RCC) have low cure rates or significant side effects. It has been reported that complexes composed of interleukin (IL)-2 and stimulating anti-IL-2 antibody (IL-2C) suppress malignant melanoma growth. We investigated whether it could have similar effects on RCC.

**Methods:**

A syngeneic RCC model was established by subcutaneously injecting RENCA cells into BALB/c mice, which were administered IL-2C or phosphate-buffered saline every other day for 4 weeks. RCC size was measured serially, and its weight was assessed 4 weeks after RENCA injection. Immune cell infiltration into RCC lesions and spleen was assessed by flow cytometry and immunohistochemistry.

**Results:**

IL-2C treatment increased the numbers of CD8^+^ memory T and natural killer (NK) cells in healthy BALB/c mice (*P* < 0.01). In the spleen of RCC mice, IL-2C treatment also increased the number of CD8^+^ memory T, NK cells, and macrophages as compared to PBS-treated controls (*P* < 0.01). The number of interferon-γ- and IL-10-producing splenocytes increased and decreased, respectively after 4 weeks in the IL-2C-treated mice (*P* < 0.01). Tumor-infiltrating immune cells including CD4^+^ T, CD8^+^ T, NK cells as well as macrophages were increased in IL-2C-treated mice than controls (*P* < 0.05). Pulmonary edema, the most serious side effect of IL-2 therapy, was not exacerbated by IL-2C treatment. However, IL-2C had insignificant inhibitory effect on RCC growth (P = 0.1756).

**Conclusions:**

IL-2C enhanced immune response without significant side effects; however, this activity was not sufficient to inhibit RCC growth in a syngeneic, murine model.

## Background

Renal cell carcinoma (RCC) is the most common primary malignancy of the renal parenchyma, comprising 3 % of all adult malignancies, and its incidence has been increasing [1, 2]. Although early RCC can be cured by surgery, one-third of RCC patients exhibit metastasis at diagnosis. Metastatic RCC has poor prognosis, with a 5-year survival rate of only 10 % [3], and approximately 20-25 % of patients with metastatic RCC do not respond to treatment and symptoms progress rapidly [4]. Sorafenib is one of target drugs against RCC that prolongs patient survival, but rarely leads to complete remission [5–7]; moreover, long-term sorafenib treatment can exacerbate RCC by creating ischemic conditions [8, 9].

RCC is considered as an immunogenic tumor owing to its spontaneous regression, variable growth, late metastasis, high degree of T cell infiltration, and high incidence in immunosuppressed patients. However, RCC can also suppress the anti-tumor immunity of naïve and memory CD4^+^ T, natural killer (NK), and dendritic cells [10], and evade the cytotoxic effect of NK cells [11, 12]. Therefore, a drug that potentiates immune response may be effective in the treatment of RCC. Indeed, high doses of interleukin (IL)-2 have been shown to suppress RCC progression without inducing tumor ischemia, leading to complete remission in 10–20 % of patients [13, 14]. Blockade of CTLA4, a T-cell inhibitory receptor with ipilimumab, and increasing T-cell proliferation and cytotoxic effects with PD-1/PD-L1 axis inhibition also induced regression of renal cell carcinoma in some patients [15, 16]. However, high-dose IL-2 therapy also induces systemic inflammatory responses, including capillary leak syndrome, heart failure, and pulmonary edema, thereby hindering the broad application of high-dose IL-2 therapy in the treatment of advanced RCC [17, 18].

Recently, immune complexes (IL-2C) composed of with low-dose IL-2 and stimulating anti-IL-2 antibody (S4B6) have been shown to enhance immune responses via selective structural interactions [19–23]. Stimulating IL-2C can preferentially expand memory CD8^+^ T and NK cells—while more weakly affecting regulatory T cells—via the interaction of anti-IL-2 antibodies (S4B6) and CD25 binding region of IL-2, leading to inhibition of both leukemia and melanoma [19, 23]. Interestingly, the half-life of IL-2 is increased in IL-2C; as such, low-dose IL-2C has immune enhancing effects that are comparable to those of high-dose IL-2 therapy without accompanying serious side effects such as capillary leak syndrome [19, 23]. Low-dose IL-2C therapy is therefore expected to be an effective and safe treatment for immunogenic tumors.

Here, we investigated the efficacy and safety of low-dose IL-2C treatment for RCC in a syngeneic murine model. We found that IL-2C treatment enhanced anti-tumor immunity against RCC without causing pulmonary edema, although it did not have sufficient potency to suppress tumor growth.

## Methods

### Cells and mice

The RENCA, a murine RCC cell line from a BALB/c mouse background was purchased from Korean Cell line Bank (Seoul, Korea), and cultured in Eagle’s Minimum Essential Medium (Gibco/Invitrogen, Grand Island, NY, USA) containing 10 % fetal bovine serum (Gibco/Invitrogen) at 37 °C and 5 % CO_2_. BALB/c mice were purchased from Orient Bio Inc. (Seongnam, Korea) and maintained at the Biomedical Research Institute of Seoul National University Hospital. Mouse experimental protocols were approved by the Animal Ethics Committee of Seoul National University College of Medicine.

### Preparation of IL-2/anti-IL-2 antibody complex

Recombinant murine IL-2 was purchased from eBioscience (San Diego, CA, USA) and the S4B6 anti-mouse IL-2 monoclonal antibodies was provided by Dr. Charles D. Surh (La Jolla Institute for Allergy and Immunology, La Jolla, CA, USA). S4B6 (7.5 μg) was mixed with IL-2 (1.5 μg, equivalent to 8555 IU) and incubated at 37 °C for 30 min before use. To evaluate the immune-enhancing effects of IL-2 under normal conditions, IL-2C or phosphate-buffered saline (PBS) was administered daily to mice by intraperitoneal injection for 5 days, before the spleen was harvested for immune cell analysis.

### In vivo tumor model

Eight-week old BALB/c mice were subcutaneously injected with RENCA cells (1 × 10^5^) in 0.1 ml of 1× PBS to induce syngeneic RCC formation. IL-2C (treatment group) or PBS (control group) was intraperitoneally administered to mice every other day from day 0 to 28. Tumor size (length × width) was measured every other day using calipers. IL-2C with S4B6 (7.5 μg) and IL-2 (1.5 μg) or phosphate-buffered saline (PBS) was administered every 2 days to mice by intraperitoneal injection until 28 days. In high-dose IL-2 group, higher dose of IL-2 (35 μg, 200,000 IU) was administered to mice with the same schedule. Spleen, lung and tumor tissues were harvested 28 days after injection of RENCA cells. Tumor weight was measured after harvest. Pulmonary edema was assessed by lung weight, which was calculated by subtracting the dry weight from the wet weight.

### Flow cytometry

Splenocytes were labeled with the following antibodies: anti-CD4-allophycocyanin (APC), anti-CD8-fluorescein isothiocyanate (FITC), anti-CD44-APC, anti-CD45-FITC, anti-CD49-phycoerythrin (PE), and anti-F4/80-PE and the vital dye 7-aminoactinomycin D (7-AAD) (BD Biosciences, San Jose, CA, USA). Forkhead homeobox protein 3 (Foxp3) was labeled using the anti-mouse Foxp3-FITC staining kit (eBioscience) according to the manufacturer’s instructions. For analysis of tumor-infiltrating cells, tumors were dissociated with 200U/ml collagenase IV at 37 °C for 30 min. Flow cytometric analysis was carried out on a Canto II Instrument (BD Biosciences).

### Enzyme-linked immunoSPOT (ELISPOT) assay

Interferon (IFN)-γ- or IL-10-producing T cells were detected with the ELISPOT assay. Spleens were harvested 28 days after mice were injected with RENCA cells. A 96-well plate was coated with anti-IFN-γ or -IL-10 capture antibodies using ELISPOT mouse IFN-γ or mouse IL-10 kits (BD biosciences). For IFN-γ ELISPOT, splenocytes (1 × 10^5^/well) were incubated with 5 ng/ml phorbol 12-myristate 13-acetate (Sigma, St. Louis, MO, USA) and 500 ng/ml of inomycin (Sigma) at 37 °C for 8 h. For IL-10 ELISPOT, splenocytes (5 x 10^5^/well) were incubated with 1 μg/ml of lipopolysaccharide (Sigma) for 24 h. Detection antibodies were then added, along with horseradish peroxidase (HRP)-streptavidin (BD Biosciences). After adding 3'-amino-9-ethylcarbazole substrate (BD Biosciences) for development, colored spots were measured with an ELSPOT reader (Cellular- Technology, Cleveland, OH, USA).

### Immunohistochemistry

Tumor tissue with overlying skin was harvested on day 28. Anti-CD4, anti-CD8, anti-CD49b and anti-F4/80 antibodies (eBioscience) were incubated with tissue sections at 4 °C overnight. Sections were then treated sequentially with secondary antibody (ZytoChem Plus HRP One-Step Polymer anti-mouse; Zytomed, Berlin, Germany) and substrate solution (ImmPACT NovaRED Peroxidase Substrate Kit; Vector, Burlingame, CA, USA). Pulmonary edema was assessed by hematoxylin and eosin staining.

### Statistical analysis

Continuous variables were compared between the IL-2C and the PBS groups using the Student’s *t*-test. RCC growth over 4 weeks was compared between the two groups with the linear mixed model. A P value < 0.050 was considered statistically significant. Analyses were carried out using SPSS v.22.0 software (SPSS Inc., Chicago, IL, USA).

## Results

### IL-2/anti-IL-2 antibody complex treatment induces the expansion of CD8^+^ memory T and NK cells in the spleen

**Fig. 1:**
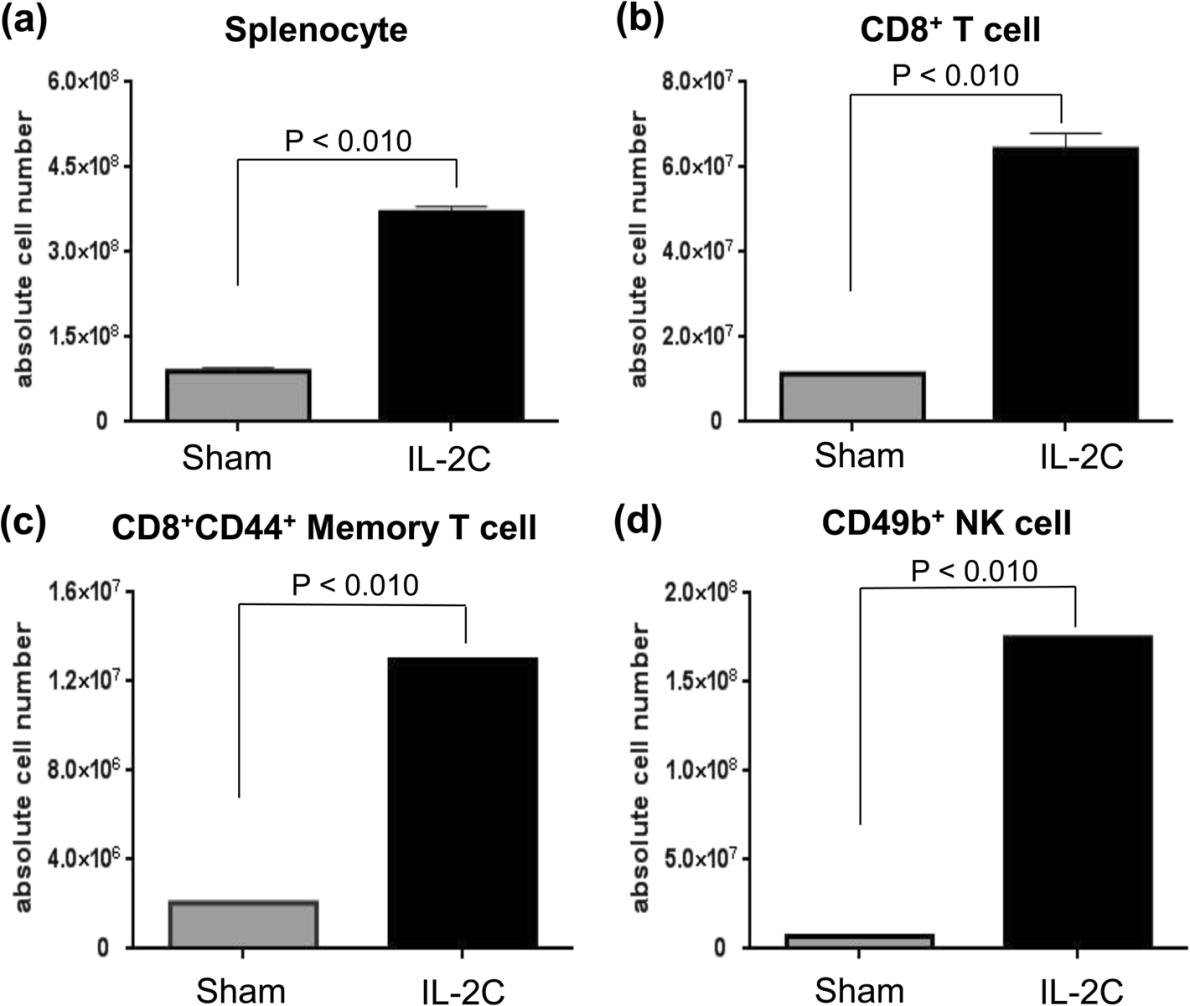
IL-2C treatment induces the expansion of CD8^+^ memory T and NK cells in the spleen. Mice were treated with IL-2C by intraperitoneal injection for 5 days. The total numbers of (**a**) splenocytes, (**b**) CD8^+^ T cells, (**c**) CD8^+^ memory T cells, (**d**) and NK cells were higher in IL-2C-treated than in PBS-treated mice (*P* < 0.010)

IL-2C were injected into healthy mice for 5 consecutive days to evaluate its immune-enhancing effects. The total numbers of splenocytes (Fig. 1a; *P* < 0.010) and CD8^+^ T cells (Fig. 1b; *P* < 0.010) were increased in the IL-2C group as compared to the PBS group. IL-2C treatment also increased the numbers of CD44^+^CD8^+^ memory T (Fig. 1c; *P* < 0.010) and CD49b^+^ NK (Fig. 1d; *P* < 0.010) cells. These results suggest that IL-2C treatment can enhance anti-tumor immunity.

### IL-2/anti-IL-2 antibody complex treatment induces the expansion of CD8^+^ memory T and NK cells in the spleen of RCC mice

**Fig. 2:**
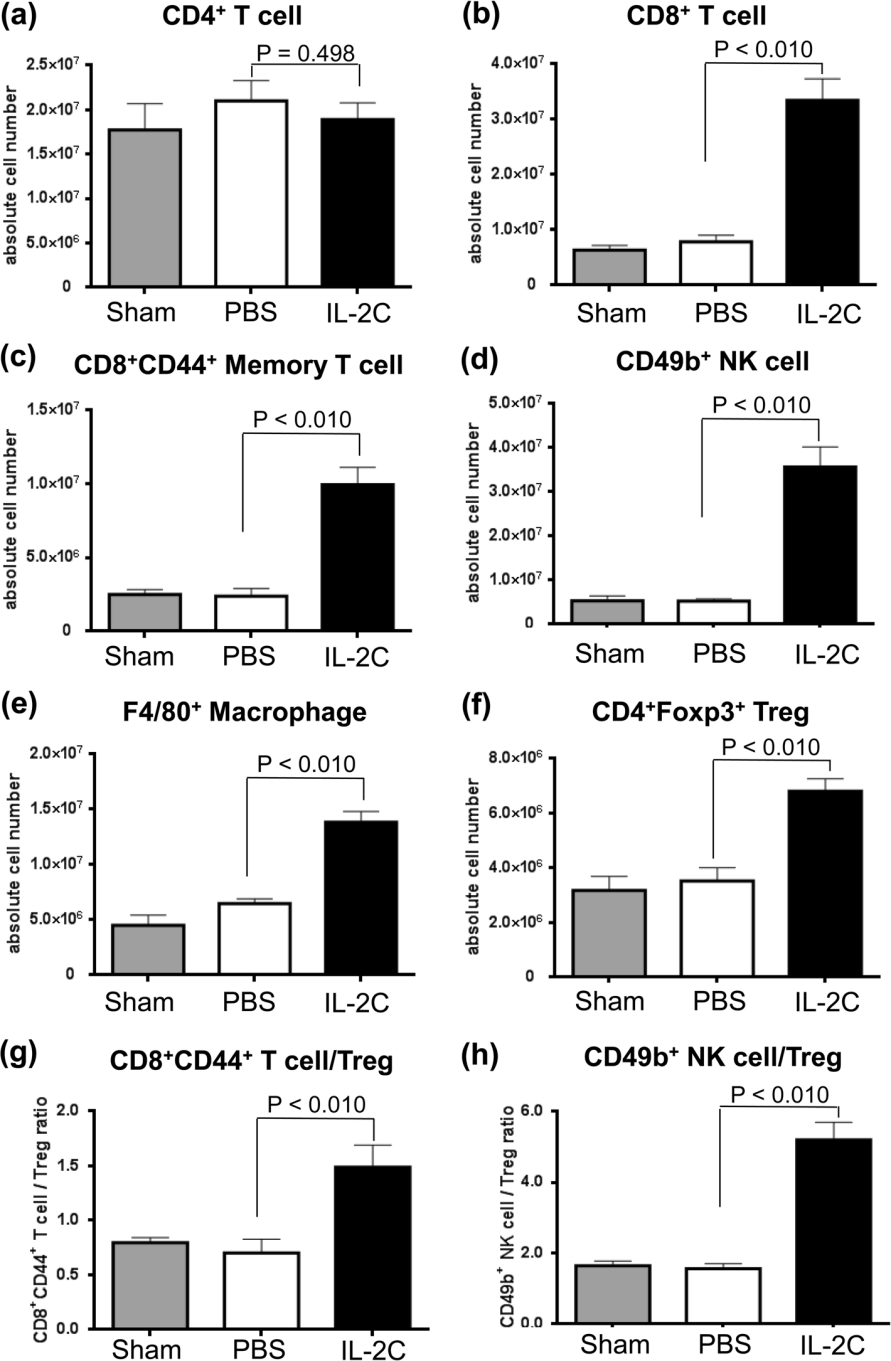
IL-2C treatment induces the expansion of immune cells in the spleen of mice with RCC. Syngeneic RENCA cells were implanted subcutaneously in mice, and IL-2C or PBS was administered every other day for 4 weeks. (**a**) The number of CD4^+^ T cells was similar between the two groups (P = 0.498), while the numbers of (**b**) CD8^+^ T cells, (**c**) CD8^+^ memory T cells, (**d**) NK cells, (**e**) macrophages, and (**f**) Tregs were higher in the IL-2C group than in the PBS group (*P* < 0.010). (**g**, **h**) CD8^+^ memory T cell/Treg and NK cell/Treg ratios were higher in the IL-2C group than the PBS group (*P* < 0.010 in both cases). Treg, regulatory T cell

Mice were subcutaneously injected with syngeneic RENCA cells, followed by IL-2C or PBS administration every other day for 4 weeks. A sham group received PBS without RENCA cell implantation. Among RCC mice, there was no difference in the number of CD4^+^ T cells between the IL-2C and PBS groups (Fig. 2a; P = 0.498). However, IL-2C treatment induced the expansion of CD8^+^ T, CD8^+^ memory T, and NK cells as well as macrophages (Fig. 2b–e; *P* < 0.010), and increased the number of splenic CD4^+^Foxp3^+^ regulatory T cells (Fig. 2f; P = 0.040), albeit to a lesser degree than for CD8^+^ memory T or NK cells. As a result, CD8^+^ memory T cell/regulatory T cell (Fig. 2g; *P* < 0.010) and NK cell/regulatory T cell (Fig. 2h; *P* < 0.010) ratios were increased in the IL-2C relative to the PBS group. These data indicate that IL-2C treatment enhances anti-tumor immunity against RCC.

### IL-2/anti-IL-2 antibody complex treatment increases IFN-γ^+^ and decreases IL-10^+^ splenocyte populations

**Fig. 3:**
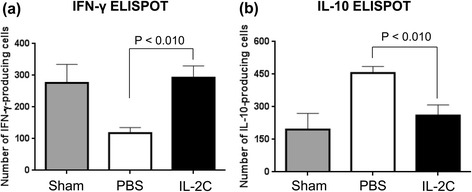
IL-2C treatment increases the number of IFN-γ^+^ splenocytes, but decreased the number of IL-10^+^ splenocytes. After syngeneic RENCA cells were implanted in mice, splenocytes were harvested on day 28 and analyzed for IFN-γ and IL-10 production by ELISPOT. **a**, **b** The number of IFN-γ-producing splenocytes was higher (**a**) but the number of IL-10-producing splenocytes were lower (**b**) in IL-2C-treated mice than in PBS-treated mice (*P* < 0.010 in both cases)

We analyzed Th1 and Th2 cytokine responses in the spleen of RCC mice (Fig. 3). The number of IFN-γ-producing splenocytes was lower in RCC mice treated with PBS than in the sham group. Meanwhile, IL-2C-treated mice had a higher number of IFN-γ^+^ splenocytes than those in the PBS group (Fig. 3a; *P* < 0.010). The number of IL-10-producing splenocytes was higher in RCC mice treated with PBS than in the sham group (*P* < 0.010), but this was decreased by IL-2C treatment (Fig. 3b; *P* < 0.01). These results indicate that IL-2C can shift the immune response from Th2 to Th1 in the RCC environment.

### IL-2/anti-IL-2 antibody complex treatment increases immune cell infiltration into RCC lesions

**Fig. 4:**
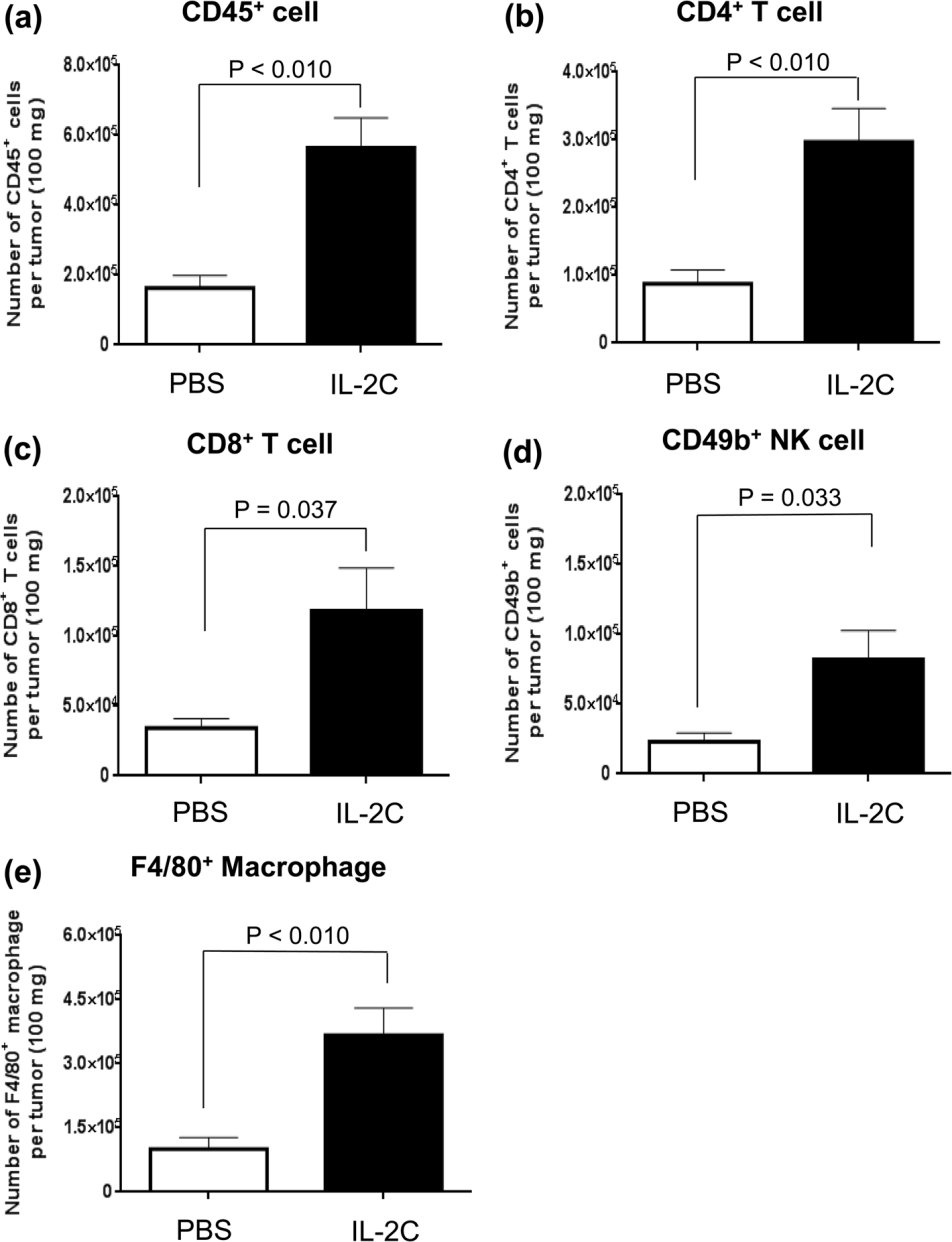
Flow cytometric analysis of immune cell infiltration into RCC lesions. Tumors were harvested on day 28 after mice were implanted with RENCA cells and immune cells were detected by flow cytometry. IL-2C treatment increased infiltration of (**a**) CD45^+^ cells (*P* < 0.010), (**b**) CD4^+^ T cells (*P* < 0.010), (**c**) CD8^+^ T cells (P = 0.037), (**d**) NK cells (P = 0.033) (**e**) and macrophages (*P* < 0.010) into tumors

**Fig. 5:**
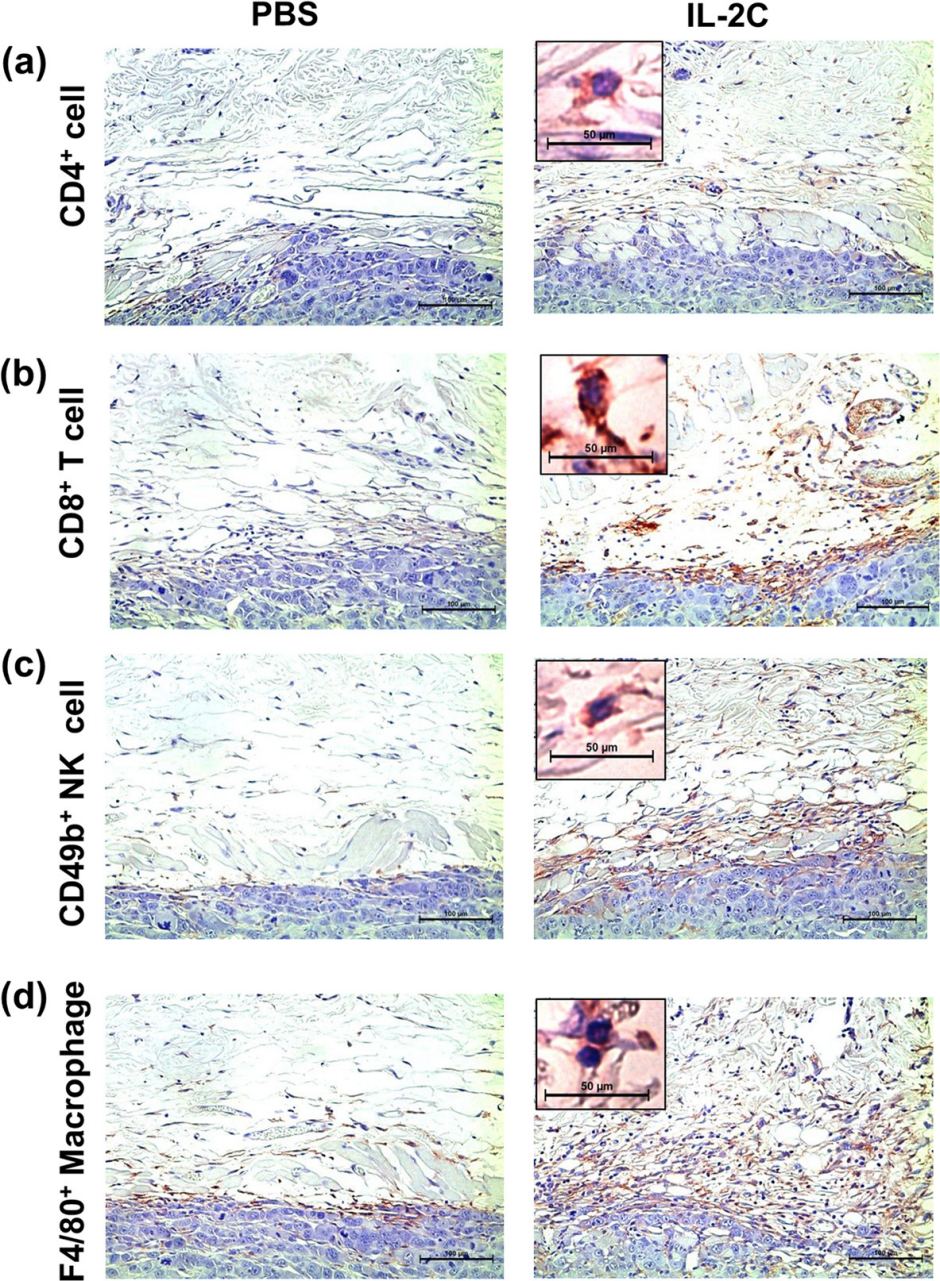
Immunohistochemical analysis of immune cell infiltration into RCC lesions. Tumors were harvested on day 28 after mice were implanted with RENCA cells and immune cells were detected by immunohistochemistry. The size of immune cell populations, including (**a**) CD4^+^ T cells, (**b**) CD8^+^ T cells, (**c**) NK cells and (**d**) macrophages along tumor margins was increased by IL-2C treatment. Images are shown at 200× magnification. Insets show immunoreactive cells at 1000× magnification

Given the immune-stimulating effects of IL-2C on the spleen in RCC, we investigated whether immune cell infiltration of immune cells into RCC lesions was induced by IL-2C treatment. Flow cytometric analysis showed higher numbers of infiltrating CD45^+^ (Fig. 4a; *P* < 0.010), CD4^+^ T (Fig. 4b; *P* < 0.010), CD8^+^ T (Fig. 4c; P = 0.037), and NK (Fig. 4d; P = 0.033) cells as well as macrophages (Fig. 4e; *P* < 0.010) in the IL-2C group than in the PBS group. In addition, an immunohistochemical analysis found that IL-2C treatment increased CD4^+^ T, CD8^+^ T, and NK cells as well as macrophages recruitment to RCC lesions (Fig. 5). However, there was no perigraft infiltration of regulatory T cells (data not shown). Taken together, these data demonstrate that IL-2C stimulates the infiltration of immune cells into RCC lesions.

### Anti-tumorigenic effects of IL-2/anti-IL-2 antibody complex were not sufficient to suppress RCC growth

**Fig. 6:**
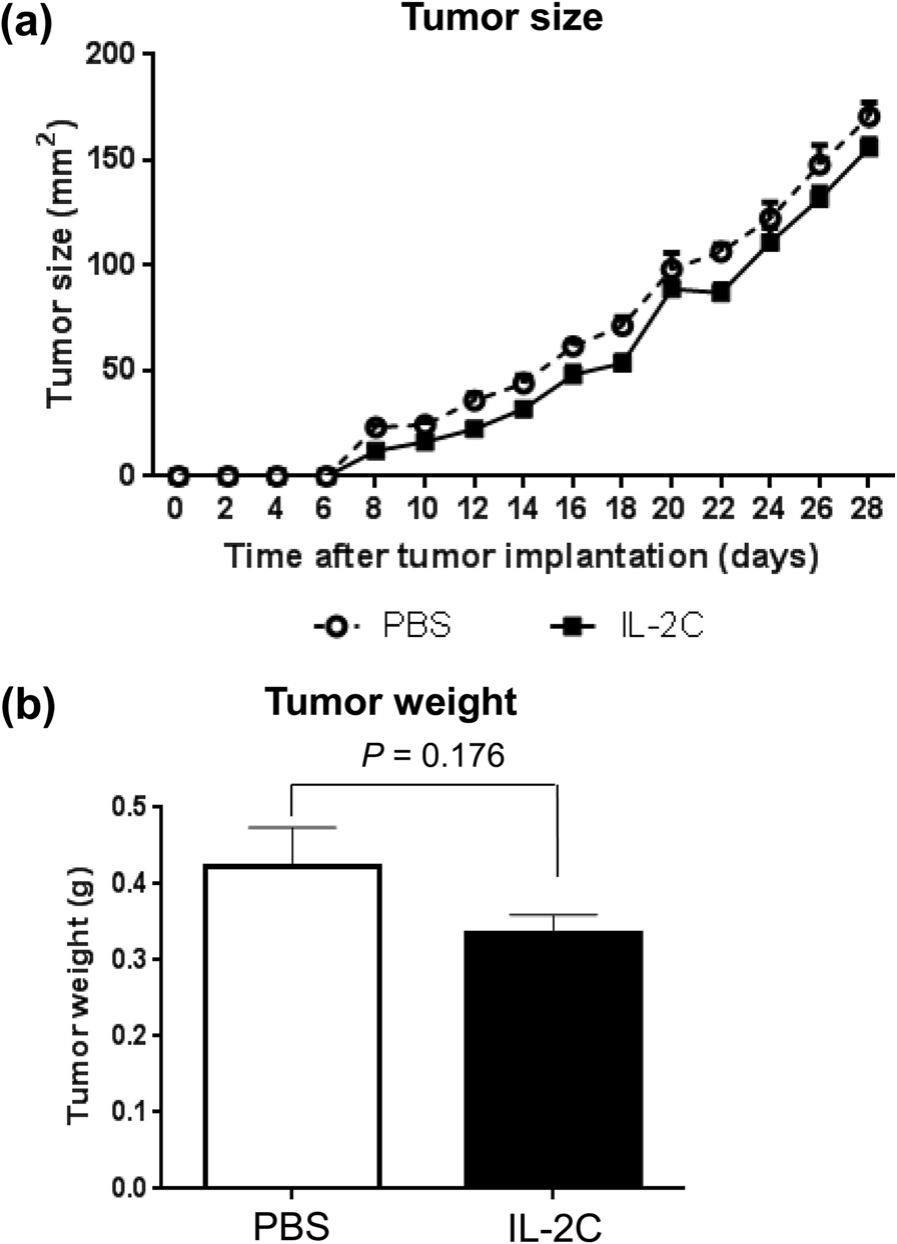
IL-2C does not suppress growth of RCC significantly. **a** IL-2C slowed the growth of syngeneic RENCA cells implanted subcutaneously into mice (P = 0.036, linear mixed model). **b** Tumor weight on day 28 did not differ significantly between the IL-2C and the PBS groups (P = 0.176, Student’s *t*-test)

The size of RCC lesions increased progressively over time in both PBS-treated and IL-2C-treated mice (Fig. 6a; *P* < 0.010); however, the rate of growth was higher in the former group (Fig. 6a; *P* = 0.036), although the difference was slight. However, tumor weights on day 28 did not differ significantly between the two groups (Fig. 6b; *P* = 0.176). These data suggest that the potentiation of anti-tumor immunity by IL-2C was not sufficient to suppress RCC growth significantly.

### IL-2/anti-IL-2 antibody complex treatment does not induce pulmonary edema

**Fig. 7:**
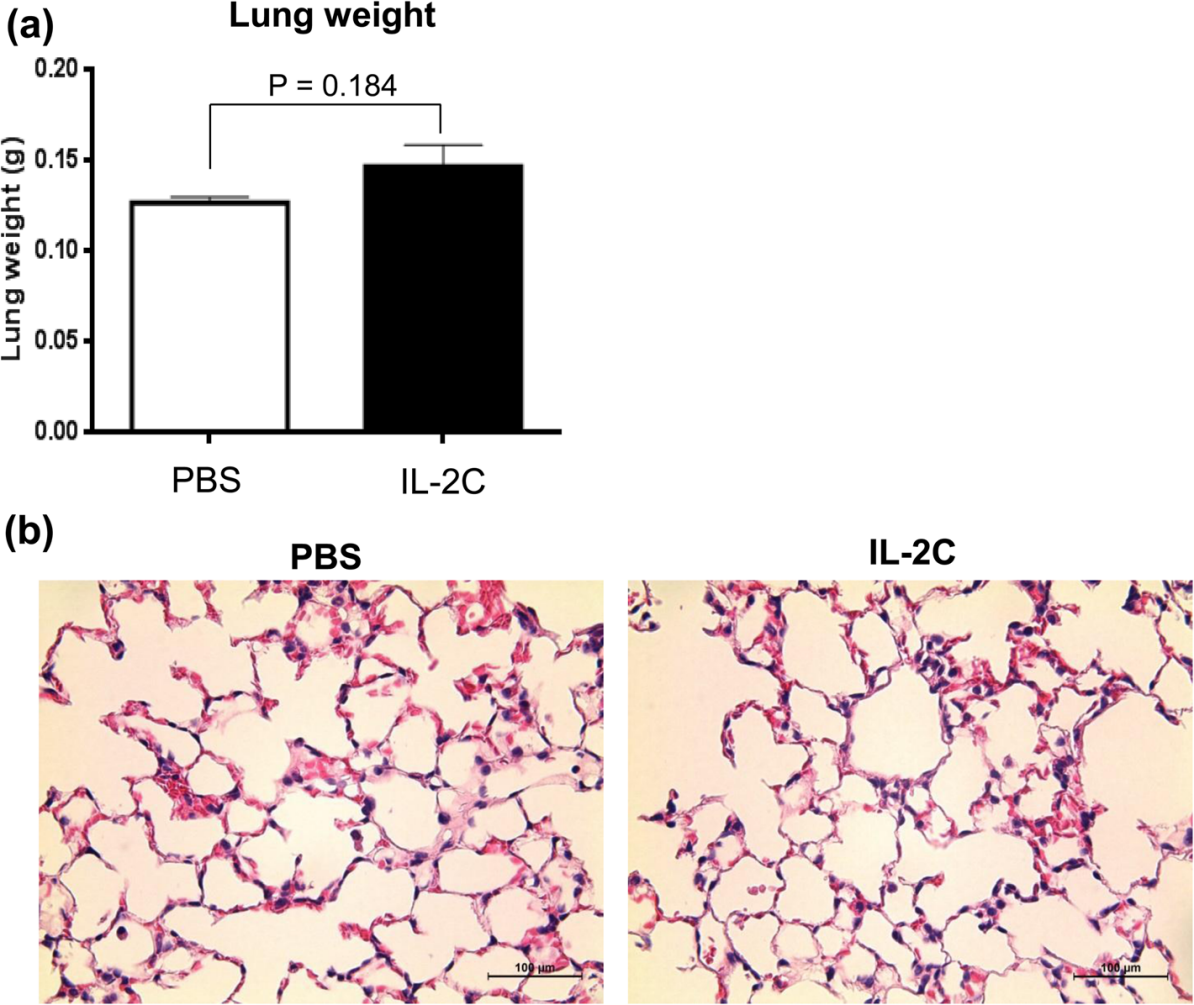
IL-2C does not exacerbate pulmonary edema in mice with RCC. Lung weight was measured by subtracting dry from wet weight immediately after harvesting on day 28. **a** Lung weight did not differ significantly between IL-2C-treated mice and PBS-treated mice (P = 0.184). **b** IL-2C treatment did not increase pulmonary edema, as visualized by hematoxylin and eosin staining. Images are shown at 400× magnification

Pulmonary edema is a manifestation of capillary leak syndrome and is the most serious side effect of high-dose of IL-2 therapy [23]*.* On day 28, there was no significant difference in lung weights between IL-2C- and PBS-treated mice (Fig. 7a; P = 0.184). A histologic examination revealed no evidence of increased pulmonary edema by IL-2C treatment (Fig. 7b). These results demonstrate that IL-2C is safe for use, as it does not carry a significant risk of pulmonary edema development.

### Comparison between IL-2/anti-IL-2 antibody complex treatment and high-dose IL-2 therapy

**Fig. 8:**
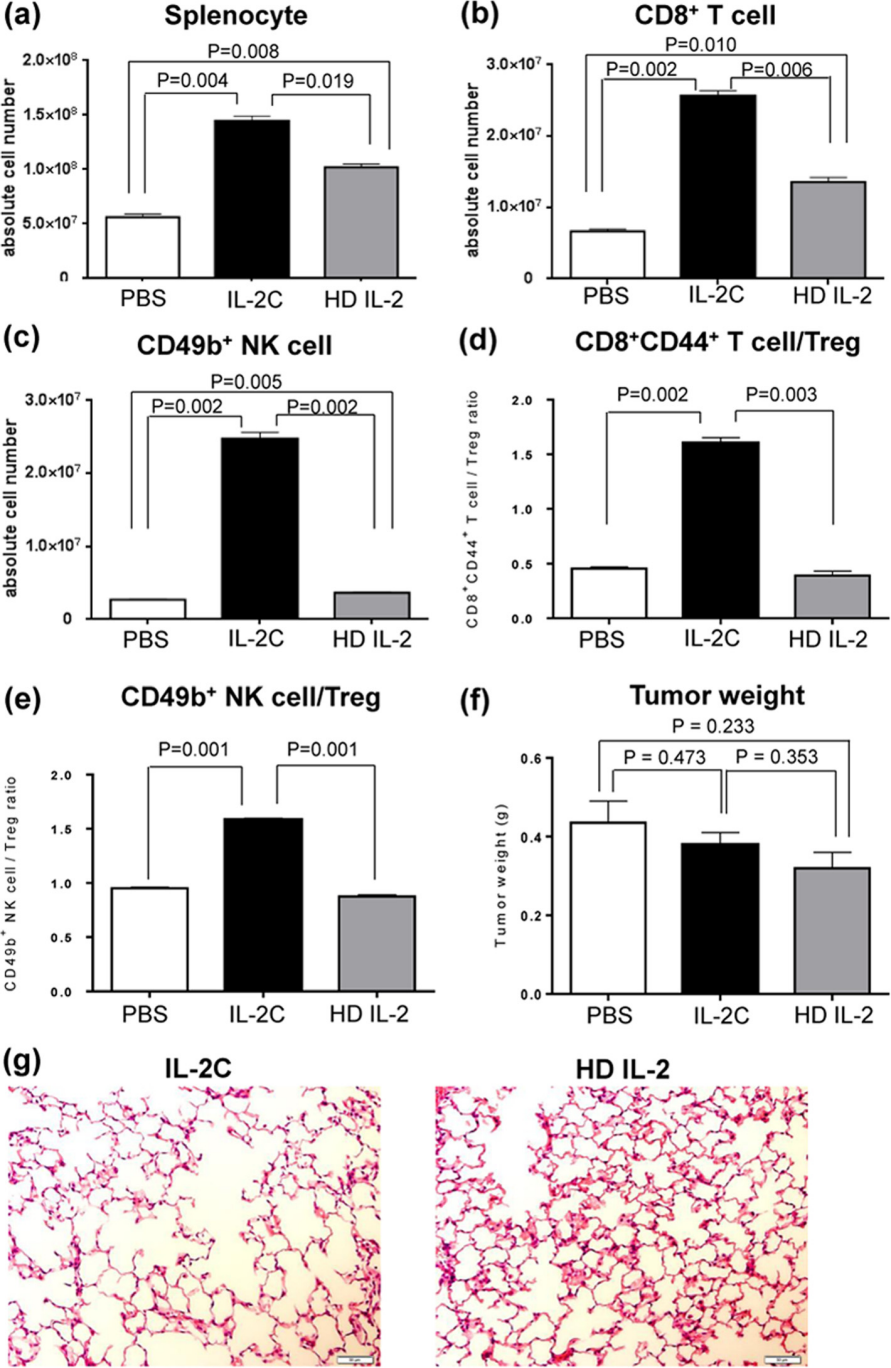
Comparison between IL-2C therapy and high-dose IL-2 therapy. IL-2C treatment induces more expansion of splenic immune cells than high-dose IL-2 therapy (**a**-**e**). **a** Both IL-2C (P = 0.004) and high-dose IL-2 (P = 0.008) increased the number of splenocytes; however, the effect of IL-2C was greater than that of high-dose IL-2 (P = 0.019). **b** CD8^+^ T cells were also increased more by IL-2C than high-dose IL-2 (P = 0.006). **c** Only IL-2C increased the number of NK cells (P = 0.002). **d**-**e** IL-2C increased both ratio of CD8^+^CD44^+^ T cells/Tregs (P = 0.002, **d**), and ratio of CD49b^+^ NK cells/Tregs (P = 0.001, **e**), whereas high-dose IL-2 did not. **f** Either IL-2C or high-dose IL-2 did not suppress growth of RCC significantly. Tumor weight on day 28 did not differ significantly between the IL-2C and the high-dose IL-2 groups (P = 0.353). **g** Pulmonary edema looked more severe in the high-dose IL-2 group than IL-2 complex group; however difference was not significant. Images are shown at 200× magnification. IL-2C, interleukin-2/anti-interleukin-2 antibody complex; HD, high dose; Treg, regulatory T cell

When immune potentiating effects of IL-2C were compared with those of high-dose IL-2 therapy, the IL-2C therapy increased total leukocytes, CD8^+^ T cells, NK cells, and macrophages in both spleen (Fig. 8) and peritumor tissues (data not shown) to greater extent than the high-dose IL-2 therapy. The ratio of either splenic CD8^+^CD44^+^ T cells/Tregs or CD49b + NK cell/Tregs were not significantly increased in the high-dose IL-2 group (Fig. 8d-e). There was no difference in RCC weight between the IL-2C group and the high-dose IL-2 group (Fig. 8f). Pulmonary edema looked more severe in the high-dose IL-2 group than IL-2 complex group (Fig. 8g); however there was no significant difference in lung weight between the two groups (*P* > 0.05). Taken together, IL-2C induced more immune potentiating effects with lesser dose than high-dose IL-2 therapy; however IL-2C did not show significant benefits in either tumor reduction or pulmonary edema in the present dose.

## Discussion

The present study investigated for the first time the anti-tumorigenic effects of IL-2C against RCC in vivo. We found that stimulating IL-2C induced the expansion of CD8^+^ memory T and NK cell populations, shifted the Th1/Th2 balance in favor of Th1, and increased immune cell infiltration into tumor tissue in mice with RCC, all without inducing serious side effects such as pulmonary edema. However, the enhancement of anti-tumor immunity by IL-2C was not sufficient to inhibit RCC growth significantly.

IL-2C can enhance or suppress immunity depending on the type of anti-IL-2 monoclonal antibody. For example, the monoclonal antibody JES6-1 binds to the IL-2 epitope, and hinders binding to IL-2 receptor (R)-β while enabling binding to IL-2R-α. Since both CD8^+^ memory T and NK cells constitutively express IL-2R-β, and regulatory T cells constitutively express both IL-2R-β and IL-2R-α, an IL-2C comprising JES6-1 preferentially induced the expansion of regulatory T cells [24]. In contrast, S4B6 binds to an epitope of IL-2 such that binding to IL-2R-α is blocked in favor of IL-2R-β binding [23]. Therefore, IL-2C comprising S4B6 induces the expansion of CD8^+^ memory T and NK cells over regulatory T cells.

Immune complexes consisting of low-dose IL-2 and the S4B6 clone of the anti-IL-2 antibody was found to inhibit metastasis of melanoma and leukemia in a mouse model by inducing the expansion of CD8^+^ T and NK cell populations [19, 23]. In accordance with these findings, we also found that S4B6-containing IL-2C increased CD8^+^ T and NK cell number as well as their infiltration into RCC lesion, although the growth of RCC was not significantly affected in a syngeneic RCC mice model.

There are a few possible explanations for the insufficient effects of IL-2C on RCC growth. Firstly, immunosuppression by RCC is strong enough to counter immune-potentiating effects of IL-2C, which promotes RCC proliferation and survival [10–12]. For instance, RCC exhibits resistance to NK cell-mediated lysis, despite IL-2C-induced NK cell expansion and infiltration into RCC lesions [11, 12]. Secondly, the immunogenicity of RCC may be lower than that of malignant melanoma. Tumor-associated antigens are required for immune cell infiltration into tumors [25, 26]; however, there are fewer RCC-associated antigens than tumor-associated antigens that have been found in melanoma [27]. Therefore, a relative lack of targeting antigens may be a reason why adoptive therapy with CD8^+^ tumor-infiltrating lymphocytes has not been clinically effective for RCC treatment [28]. Third, lack of kidney-specific microenvironment might have influenced the results. However, when we injected RENCA cells into the renal subcapsular space, the results were the same as those in the subcutaneous RCC model (data not shown).

The amount of IL-2 that was used in IL-2C therapy was 23 times lower than the amount of IL-2 in high-dose IL-2 therapy [23]. Based on a previous report [23] and our results, low-doses of IL-2C do not cause significant adverse reactions such as pulmonary edema, and is therefore safe for clinical application. However, because even high-dose IL-2 therapy in the present study did not increase lung weight significantly, further studies using higher dose of IL-2C and IL-2 are needed to confirm safety as well as insufficient efficacy of IL-2C in comparison to high-dose IL-2.

Since IL-2C alone cannot suppress RCC growth, additional studies are needed to determine the impacts of other therapies used in combination with IL-2C on RCC. For example, IL-15 can also induce the expansion of NK and CD8^+^ T cell populations and thereby suppress the growth of malignant melanoma [29], and a complex of IL-15 and soluble IL-15Rα has even more potent effects [30]. Therefore, it is worth investigating whether IL-2C used in conjunction with an IL-15 complex has greater effectiveness in suppressing RCC growth. We may also try to combine IL-2C with the current target agents such as sorafenib to obtain additive effects.

## Conclusions

Stimulating IL-2C treatment potentiated anti-tumor immunity without causing significant side effects; however, given that the immune-enhancing effects of IL-2C were not sufficiently strong to suppress RCC growth, its use in combination with other therapy should be considered.

## Abbreviations

7-AAD: 7-aminoactinomycin D
APC: Allophycocyanin
FITC: Fluorescein isothiocyanate
Foxp3: Forkhead homeobox protein 3
IFN: Interferon
IL: Interleukin
IL-2C: Complex of interleukin (IL)-2 and stimulating anti-IL-2 antibody
NK cell: Natural killer cell
PBS: Phosphate-buffered saline
PE: Phycoerythrin
RCC: Renal cell carcinoma
RENCA: Murine renal cell carcinoma.
